# Impact of a 6-week non-energy-restricted ketogenic diet on physical fitness, body composition and biochemical parameters in healthy adults

**DOI:** 10.1186/s12986-017-0175-5

**Published:** 2017-02-20

**Authors:** Paul Urbain, Lena Strom, Lena Morawski, Anja Wehrle, Peter Deibert, Hartmut Bertz

**Affiliations:** 1 Department of Medicine I, Section of Clinical Nutrition and Dietetics, Medical Center – University of Freiburg, Faculty of Medicine, University of Freiburg, Hugstetterstr 55, 79106 Freiburg, Germany; 2 Institute of Agricultural and Nutritional Sciences, Martin-Luther-University Halle-Wittenberg, Halle, Germany; 3 Department of Sport and Sport Science, University of Freiburg, Freiburg, Germany; 4 Institute for Exercise- und Occupational Medicine, Center for Medicine, Medical Center – University of Freiburg, Faculty of Medicine, University of Freiburg, Freiburg, Germany; 5 Department of Medicine I, Hematology, Oncology and Stem Cell Transplantation, Medical Center – University of Freiburg, Faculty of Medicine, University of Freiburg, Freiburg, Germany

**Keywords:** Ketogenic diet, Low carbohydrate, Non-energy-restricted diet, Endurance capacity, Cardiopulmonary exercise testing, Strength, Physical performance, Blood lipids, Body composition

## Abstract

**Background:**

The ketogenic diet (KD) is a very low-carbohydrate, high-fat and adequate-protein diet that without limiting calories induces different metabolic adaptations, eg, increased levels of circulating ketone bodies and a shift to lipid metabolism. Our objective was to assess the impact of a 6-week non-energy-restricted KD in healthy adults beyond cohorts of athletes on physical performance, body composition, and blood parameters.

**Methods:**

Our single arm, before-and-after comparison study consisted of a 6-week KD with a previous preparation period including detailed instructions during classes and individual counselling by a dietitian. Compliance with the dietary regimen was monitored by measuring urinary ketones daily, and 7-day food records. All tests were performed after an overnight fast: cardiopulmonary exercise testing via cycle sprioergometry, blood samples, body composition, indirect calorimetry, handgrip strength, and questionnaires addressing complaints and physical sensations.

**Results:**

Forty-two subjects aged 37 ± 12 years with a BMI of 23.9 ± 3.1 kg/m^2^ completed the study. Urinary ketosis was detectable on 97% of the days, revealing very good compliance with the KD. Mean energy intake during the study did not change from the habitual diet and 71.6, 20.9, and 7.7% of total energy intake were from fat, protein, and carbohydrates, respectively. Weight loss was −2.0 ± 1.9 kg (*P* < 0.001) with equal losses of fat-free and fat mass. VO_2_peak and peak power decreased from 2.55 ± 0.68 l/min to 2.49 ± 0.69 l/min by 2.4% (*P* = 0.023) and from 241 ± 57 W to 231 ± 57 W by 4.1% (*P* < 0.001), respectively, whereas, handgrip strength rose slightly from 40.1 ± 8.8 to 41.0 ± 9.1 kg by 2.5% (*P* = 0.047). The blood lipids TG and HDL-C remained unchanged, whereas total cholesterol and LDL-C increased significantly by 4.7 and 10.7%, respectively. Glucose, insulin, and IGF-1 dropped significantly by 3.0, 22.2 and 20.2%, respectively.

**Conclusions:**

We detected a mildly negative impact from this 6-week non-energy-restricted KD on physical performance (endurance capacity, peak power and faster exhaustion). Our findings lead us to assume that a KD does not impact physical fitness in a clinically relevant manner that would impair activities of daily living and aerobic training. However, a KD may be a matter of concern in competitive athletes.

**Trial registration:**

DRKS00009605, registered 08 January 2016.

**Electronic supplementary material:**

The online version of this article (doi:10.1186/s12986-017-0175-5) contains supplementary material, which is available to authorized users.

## Background

The ketogenic diet (KD) is a very low-carbohydrate (<10% of energy), high-fat (>60% of energy) and adequate-protein diet that without limiting calories induces a metabolic condition called “physiological ketosis” involving increased levels of circulating ketone bodies [1]. The KD is a long-time proven therapy for intractable childhood epilepsy [2]. Its therapeutic use in a range of diseases such as type 2 diabetes, polycystic ovary syndrome, neurodegenerative diseases, and cancer is currently being investigated [3]. Moreover, KDs recently have become quite popular as a weight-loss diet [4].

According to recently published pilot studies, KDs appear to be safe and feasible in cancer patients [5, 6]. Although the current scientific evidence does not justify recommending a KD in cancer patients, a growing number of cancer patients put themselves on a KD at their own risk, outside of clinical trials, and often without medical supervision in response to frequent press reports and health books. Regular physical exercise during and after anti-cancer therapy plus adequate nutrition result in improvements in physical functioning, quality of life, and may reduce cancer-related fatigue [7] as well as malignant recurrences and mortality among several cancers [8–10]. Any diet that potentially compromises physical performance and someone’s capacity to adhere to an exercise regime would be of great concern.

An analysis of the existing literature of the KD’s effects on endurance and physical performance was conducted, excluding those studies using carbohydrate-restricted diets unable to produce ketosis (carbohydrate intake >50 g/day), hypocaloric diets, and any intervention periods lasting under a week. We identified only four small studies meeting our search criteria [11–14], whereas three included performance athletes [11–13] and one applied a high-protein, probably non-ketogenic diet [12]. Hence, the available data is very limited, thus our objective was first to assess in a larger trial the impact of a non-energy-restricted, 6-week KD in healthy adults beyond cohorts of performance athletes on physical performance (endurance capacity and muscle strength), body composition, and a range of blood parameters.

## Methods

### Subjects

Adults in good general health with a body mass index (BMI) in the range of 19–30 kg/m^2^ were recruited from employees of the University Medical Center Freiburg and their family and friends via advertising from February to June 2016. Exclusion criteria included low-carbohydrate nutrition, impaired liver and renal function, kidney stones, pregnancy or lactation period, diabetes mellitus, and any fatty acid-metabolism disorders. The study protocol was approved by the Ethics Commission of Albert-Ludwig University Freiburg (494/14) and all subjects signed a written consent form. The study was registered at germanctr.de as DRKS00009605.

### Study design and intervention

This study had a single arm before-and-after comparison design. The experimental intervention consisted of a KD without caloric restriction lasting 6 weeks (42 days) with a previous preparation period including detailed instructions during classes and individual counselling by a dietitian. Day one and day 42 will subsequently be denoted PRE and POST, respectively.

Our dietary recommendations and handouts were similar to those used in the study by Klement et al. [14]. Our subjects were provided with handouts summarizing the main aspects of a KD and given a list of suitable foods with very low carbohydrate content. Furthermore, the subjects shared cooking recipes and links to helpful webpages on an internal weblog. They were free to follow a KD according to their personal preferences but were advised to eat *ad libitum* but limit their carbohydrate intake to a maximum of 20–40 g/day to derive at least 75%, 15–20%, and 5–10% of total energy from fats, protein, and carbohydrates, respectively. In the first intervention week the subjects were instructed to switch in a gradual and well-controlled manner from their usual to a KD with the objective to attain stable ketosis by the end of the week, as such transition periods can be accompanied by short-term side effects including gastrointestinal symptoms (eg, constipation) and slight headache [15]. The subjects received a logbook to record daily any side effects and complaints during the KD intervention. To avoid biasing the cardiopulmonary exercise testing at POST, the subjects were advised not to alter their physical activities during the study period. Physical activity was assessed at PRE and POST using a validated questionnaire developed by Frey et al. [16].

Compliance with the dietary regimen was monitored by taking daily measurements of urinary ketones and keeping 7-day food records. The subjects documented their daily urinary ketone measurements (acetoacetate) using self-testing strips (Ketostix, Bayer Vital GmbH, Leverkusen, Germany). An initial substudy revealed that ketonuria can be most reliably detected in the early morning and post-dinner urine [17]. Those results also enabled our dietitian to individually fine-tune their diets if necessary via phone or personal contact, thus ensuring continuous ketosis.

Two semi-quantitative 7-day food records were obtained from all subjects before and during the last week of the intervention. Our dietitian gave them precise oral and written instructions individually on how to accurately record the amounts and types of food and beverages. Subjects were given a digital portable scale (KS 22, Beurer GmbH, Ulm, Germany) and instructed to weigh all food items separately if possible or to estimate the amounts and take a photograph. The energy, macro- and micronutrient intakes were analysed with a nutritional database software (Prodi 6.5 basis, Nutri-Science GmbH, Stuttgart, Germany).

### Testing procedure

All testing procedures were performed at the Institute for Exercise- and Occupational Medicine in the morning between 07:00 and 09:30 after an overnight fast lasting at least 8 h. The subjects were not allowed to exercise the day before, and were advised to arrive to the examinations without any physical effort. Our endpoints are hereafter described in the chronological order recorded at PRE and POST.

Venous blood was drawn and the tubes sent immediately to our Institute for Clinical Chemistry and Laboratory Medicine. All parameters tested are listed in Table 4. Height to the nearest 1 cm was measured at PRE using a wall-mounted stadiometer. Fat mass (FM) and fat-free mass (FFM) were determined via air displacement plethysmography (ADP) using the BodPod device (Cosmed USA Inc., California, USA), which was calibrated prior to each use according to the manufacturer’s guidelines. The subjects (wearing tight-fitting underwear and a bathing cap) were weighed using the device’s corresponding scale (Tanita Corp., Tokyo, Japan). As many ADPs were performed as necessary to obtain two body volume measurements within 150 ml.

The respiratory exchange ratio (RER) at rest served to estimate the respiratory quotient (RQ). An airtight mask covering nose and mouth was used for measuring respiratory gases for 20 min with the spirometer MetaLyzer 3B-R3 (Cortex Biophysik GmbH, Leipzig, Germany) while the subjects were in supine position in a quiet and darkened atmosphere and a thermoneutral environment (24–26 °C). The RER was measured at a steady-state interval of 5 min and used to calculate 24-h resting energy expenditure (REE) using the modified Weir equation [18]. Subjects then underwent a 12-lead electrocardiogram (ECG) via the Custo diagnostic device (Custo med GmbH, Ottobrunn, Germany). Still in supine position, body compartments FM, FFM and body cell mass were determined via bioelectrical impedance analysis BIA 2000-M (Data Input, Pöcking, Germany) following a standardized procedure according to guidelines [19].

Maximum incremental cycling test was performed on an electronically braked cycle ergometer (ergoline 100, Ergoline GmbH, Bitz, Germany) with continuous monitoring of ECG, heart frequency, and blood pressure. Gas exchange and ventilation were recorded continuously via breath-by-breath gas analysis (MetaLyzer 3B-R3, Cortex Biophysik GmbH, Leipzig, Germany), which was calibrated according to the manufacturer’s instructions prior to each test. The cycle exercise test was conducted using a ramp protocol: after a 1-min resting period, the exercise test started at a workload of 25 W and the load was increased gradually by 25 W/min until exhaustion accompanied with verbally encouragement. The following cycling test indices were determined: peak oxygen uptake (VO_2_peak), VO_2_peak adjusted for body weight (relative VO_2_peak), ventilatory threshold (VT), peak power (Pmax), maximum heart rate (HRmax), and maximum RER (RERmax). After cycling, the subjects rated their perceived exertion via the 20-point Borg scale [20].

Next, we took isometric handgrip strength measurements on the dominant hand by an electronic Digimax dynamometer (Mechatronic GmbH, Hamm, Germany) connected to a computer running the ISO-Check software version 1.1. The most comfortable distance from the handles was noted and applied at the PRE and POST tests. The testing position recommended by the American Society of Hand Therapists was used [21]. Three attempts lasting 5 s each were made, with a 30-s rest between each. The subjects were verbally encouraged and able to follow the course of their strength measurements on a screen. Their highest values were used for analysis. At POST, the subjects filled out a short non-validated questionnaire addressing several aspects of their subjective sensations during the KD.

### Sample size calculation and statistics

Reference data indicate that the difference in the response of matched pairs is normally distributed with an absolute VO_2_max standard deviation of 0.46 [22]. If the true difference in the mean at PRE and POST of matched pairs is 0.216 (10%) and assuming equal variances, we would need to study 38 subjects with a power of 80% with a one-sided *t*-test at a significance level of 5%. Considering potential drop-outs (20%), we aimed to enrol 46 subjects. All variables were tested for normal distribution (Kolmogorow-Smirnow test). Normally distributed variables are presented as means ± standard deviations and paired *t*-test was used to compare PRE and POST means. Not normally distributed variables are presented as median (minimum - maximum) and Wilcoxon rank-sum test was used. Statistical significance was set at *P* <0.05. The data were analysed using IBM SPSS 22 for statistical analysis (IBM, New York, USA).

## Results

### Characteristics of the subjects

**Fig. 1:**
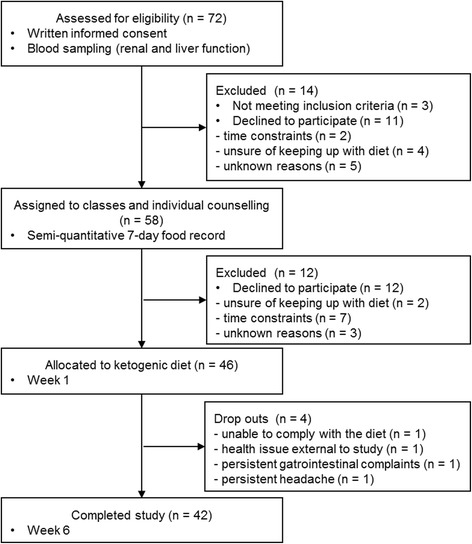
Flow diagram of the study participants from eligibility criteria screening to study completion

Out of the 72 volunteers assessed for eligibility, 46 were allocated to the KD intervention (Fig. 1). Four (8.7%) dropped out during the intervention due to persistent side effects (*n* = 2), inability to comply with the diet (*n* = 1) or a health issue (*n* = 1). The persistent reported side effects were gastrointestinal complaints and headache, common side effects that often occur during the first week [1] and that disappeared after the two subjects returned to their usual diet. Forty-two completed the 6-week intervention study, which lasted a total 42 (41–43) days. Characteristics of the study population are summarised in Table 1. Mean age and body mass index were 37 ± 12 (24–63) years and 23.9 ± 3.1 (19.0–30.4) kg/m^2^, respectively. Women made up 73.8% of the population and 88.1% reported consuming a meat-based diet. Thirteen (31.0%) subjects took medications and the most frequent therapies/drugs were thyroid (*n* = 5) and menopausal (*n* = 2) hormone replacement therapies and antidepressants (*n* = 3). The PRE questionnaire of physical activity revealed that our cohort included subjects with sedentary to vigorously active lifestyles [34.6 (8.5–156.1) MET-hours/week] and with a median physical activity level comparable to the general population [23].

**Table 1:** Characteristics of the subjects (*n* = 42)

	Unit	
Male:female	*n* (%)	11 (26.2):31 (73.8)
Age	years	37 ± 12 (24–63)
Weight	kg	70.3 ± 11.5
BMI	kg/m^2^	23.9 ± 3.1 (19.0–30.4)
Current smoker	*n* (%)	2 (4.8)
Nutrition	*n* (%)	
Meat-based		37 (88.1)
Flexitarian^a^		5 (11.9)
Medication	*n* (%)	
Thyroid hormone (L-thyroxine)		5 (11.9)
Hormone replacement therapy		2 (4.8)
Antidepressant (NDRI or SSRI)		2 (4.8)
Proton pump inhibitor		1 (2.4)
Oral anticoagulant and statins		1 (2.4)
Methylphenidate and antidepressant (NDRI)		1 (2.4)
Glucocorticoid^b^		1 (2.4)

*Abbreviations: BMI* body mass index, *NDRI* norepinephrine-dopamine reuptake inhibitor, *SSRI* selective serotonin reuptake inhibitor

^a^A plant-based diet with the occasional inclusion of meat products

^b^inhaled glucocorticoid (daily 250 μg fluticasone)

### Compliance and diet compositions

Urinary ketosis was detectable already after 2 (1–7) days from the start of the KD intervention. After the 1-week transition phase ketosis was detectable on 97% (69–100%) of the days, revealing very good compliance with the KD. Furthermore, the significant decrease in RER at rest from 0.86 to 0.79 (*P* <0.001) confirmed the metabolic shift to fat oxidation (Table 2).

**Table 2:** Physical performance and physiological parameters (indirect calorimetry, cycling test, handgrip strength)

	Unit	PRE	POST	*P*-value
Indirect calorimetry				
REE	kcal/day	1523 ± 329	1430 ± 338	0.038
RERrest		0.86 (0.75–1.14)	0.79 (0.69–0.85)	<0.001
Spiroergometry, EKG and Borg scale				
VO_2_peak (absolute)	l/min	2.55 ± 0.68	2.49 ± 0.69	0.023
VO_2_peak (relative)	ml/min*kg	36.7 ± 8.5	36.8 ± 9.0	0.808
VT1	l/min	1.23 (0.79–2.83)	1.19 (0.73–2.69)	0.233
Pmax	W	241 ± 57	231 ± 57	<0.001
HRmax	1/min	176 ± 14	177 ± 14	0.268
HRrest	1/min	57 ± 7	59 ± 7	0.008
Spinning speed	rpm	60 (58–65)	60 (58–64)	0.274
Rating of perceived exertion		17 (13–19)	18 (14–19)	0.052
Questionnaires				
Physical activity^a^	kcal/week	2575 (615–12915)	2464 (625–13760)	0.457
	MET-hours/week	34.6 (8.5–156.1)	36.7 (8.5–170.4)	0.264
Handgrip strength				
Maximum strength	kg	40.1 ± 8.8	41.0 ± 9.1	0.047

*Abbreviations: HR* heart rate, *MET* metabolic equivalent of task, *VO*_*2*_ oxygen consumption, *Pmax* Peak power, *REE* 24-h resting energy expenditure, *RER* respiratory exchange ratio, *rpm* revolutions per minute, *VT* ventilatory threshold, *W* watt

^a^Physical activity is expressed by the rate of energy expenditure in MET based on the reference data published by Ainsworth et al. [25]. One MET for a reference adult is approximately 1 kcal/kg*h

Macro- and micronutrient compositions from 7-day food records of their habitual diets and KD are shown in the Additional file 1: Table S1-S2. The mean daily caloric intake during the study did not change from the previous habitual diet (PRE 2321 ± 551 kcal, POST 2224 ± 584 kcal; *P* = 0.186) proving a non-energy-restricted diet intervention. The intake of all three macronutrients changed significantly (*P* <0.001) with higher fat and protein combined with lower carbohydrate consumption, respectively. The subjects consumed on average a KD with 71.6, 20.9, and 7.7% of total energy intake from fat, protein and carbohydrate, respectively. The ratio of the essential linoleic acid [LA, 18:2(n-6)] and alpha-linolenic acid [ALA, 18:3(n-3)] decreased significantly and improved from 8:1 to 6:1 (*P* = 0.003), coming close to the recommended ratio 5:1 [24]. In addition, the intake of 12 of the 21 evaluated vitamins and minerals during the KD changed significantly compared to the habitual diet.

### Performance tests

Cycling test and handgrip strength results are presented in Table 2. The physical activity questionnaire confirmed that subjects maintained their usual physical activity during the study. Our primary endpoint absolute VO_2_peak decreased significantly from 2.55 ± 0.68 l/min to 2.49 ± 0.69 l/min (*P* = 0.023) by 2.4%, but there was no change when expressed per kg of body weight (relative VO_2_peak). In addition, Pmax decreased significantly from 241 ± 57 W to 231 ± 57 W by 4.1%, combined with a slightly increased rating of perceived exertion [PRE 17 (13–19), POST 18 (14–19); *P* = 0.052]. VT1 and HRmax were, however, unaffected by the KD intervention. Handgrip strength increased slightly from 40.1 ± 8.8 to 41.0 ± 9.1 kg (*P* = 0.047) by 2.5%.

### Weight and body composition

We observed a significant mean body weight loss of −2.0 ± 1.9 kg ranging from −7.1 to +1.1 kg with equally significant losses of FM and FFM via ADP by −1.0 ± 1.7 kg and −1.0 ± 1.0 kg, respectively (Table 3, *P* <0.001). BIA FFM, however, did not change significantly [PRE 47.3 (38.6–76.1), POST 46.7 (38.3–75.3); *P* = 0.182] and BIA body cell mass, reflecting muscle mass, remained constant.

**Table 3:** Weight and body composition

	Unit	PRE	POST	*P*-value
Weight	kg	70.3 ± 11.5	68.4 ± 10.3	<0.001
Whole-body air displacement plethysmography (ADP)				
FM	kg	22.6 ± 8.7	21.7 ± 8.2	<0.001
FFM	kg	44.7 (36.7–72)	43.9 (36.1–70.5)	<0.001
Bioelectrical impedance analysis (BIA)				
FM	kg	20.9 ± 6.9	19.4 ± 6.3	<0.001
FFM	kg	47.3 (38.6–76.1)	46.7 (38.3–75.3)	0.182
Body cell mass	kg	24.3 (20.0–43.3)	24.4 (19.0–43.4)	0.427
Phase angle^a^	°	6.1 (4.8–8.5)	6.3 (4.8–8.2)	0.030

*Abbreviations: FFM* fat-free mass, *FM* fat mass

^a^Phase angle, one of the raw data obtained at a frequency of 50 kHz

Radian, Unit system SI derived unit, Unit of Angle, Symbol rad or °, Symbol for the unit of bioelectrical phase angle is °

### Blood parameters

Fourteen of 33 (42.4%) blood parameters changed significantly after a 6-week KD (Table 4). The blood lipids triglyceride (TG) and HDL-C remained unchanged, whereas total cholesterol (TC) (PRE 186.5 ± 34.7, POST 195.3 ± 34.7; *P* = 0.019) and LDL-C (PRE 110.9 ± 31.3, POST 122.8 ± 33.6; *P* = 0.001) increased significantly by 4.7 and 10.7%, respectively. The PRE and POST LDL-C/HDL-C ratios remained within the reference range for all subjects and did not change significantly after a 6-week KD. The TG/HDL-C ratio improved significantly by 13.5% at the end of the KD (*P* = 0.039). Blood glucose, insulin, and IGF-1 dropped significantly by 3.0, 22.2 and 20.2%, respectively. The thyroid hormone free triiodothyronine (fT3) decreased significantly by 16.3% and free thyroxine (fT4) rose by 5.5%, whereas the pituitary TSH concentration remained unchanged.

**Table 4:** Fasting blood parameters

	Unit	PRE	POST	Reference range	*P*-value
Glucose and lipids					
Glucose	mg/dl	91.4 ± 7.3	88.7 ± 5.3	74–106	0.009
TG	mg/dl	64 (38–212)	62 (39–172)	<150	0.089
HDL-C	mg/dl	71.3 ± 14.3	73.6 ± 15.6	>40	0.088
LDL-C	mg/dl	110.9 ± 31.3	122.8 ± 33.6	<160	0.001
TC	mg/dl	186.5 ± 34.7	195.3 ± 34.7	<200	0.019
LDL-C/HDL-C		1.47 (0.50–3.62)	1.58 (0.56–2.92)	<4.5	0.084
TG/HDL-C		0.89 (0.43–4.24)	0.76 (0.48–3.37)	<2	0.039
Hormones					
TSH	μU/ml	1.81 (0.01–7.26)	1.87 (0.15–10.29)	0.27–4.20	0.202
fT3	pmol/l	4.91 (3.84–10.87)	4.11 (2.91–8.80)	3.4–6.8	<0.001
fT4	pmol/l	15.7 (11.9–37.1)	16.4 (12.6–36.2)	10.6–22.7	0.008
Insulin	pmol/l	55.3 ± 23.7	43.0 ± 19.7	18–173	0.001
IGF-1	ng/ml	175 (52–427)	140 (31–337)	72–457	<0.001
Liver and kidney parameters					
CRP	mg/l	0 (0–11)	0 (0–21)	<5	0.327
Albumin	g/dl	4.51 ± 0.24	4.55 ± 0.24	3.5–5.2	0.215
GOT	U/l	21 (12–43)	23 (12–51)	10–50	0.410
GPT	U/l	19 (12–37)	22 (12–57)	10–50	0.012
Alkaline Phosphatase	U/l	56.3 ± 17.0	52.1 ± 15.3	35–130	0.001
Total bilirubin	mg/dl	0.5 (0.2–2.2)	0.4 (0.2–1.4)	<0.9–1.4	0.088
Creatinine	mg/dl	0.89 ± 0.13	0.88 ± 0.15	0.51–1.17	0.185
Urea	mg/dl	30.3 ± 8.8	34.9 ± 9.3	12.8–42.8	<0.001
Uric acid	mg/dl	4.1 (2.9–7.0)	4.5 (2.3–8.1)	2.4–7	0.001
Micronutrients					
Potassium	mmol/l	4.4 (3.6–5.4)	4.3 (3.8–5.2)	3.5–5.1	0.180
Calcium	mg/dl	2.34 ± 0.08	2.33 ± 0.07	2.15–2.50	0.411
Magnesium	mmol/l	0.82 ± 0.05	0.82 ± 0.06	0.66–1.07	0.721
Iron	μg/dl	100.9 ± 50.0	77.3 ± 32.2	37–158	0.002
Folic acid	ng/ml	7.81 ± 3.32	10.04 ± 3.92	4.6–18.7	<0.001
Blood count					
Leucocytes	10^3^/μl	5.73 (3.44–11.07)	5.66 (2.90–11.58)	3.9–10.4	0.457
Thrombocytes	10^3^/μl	255.8 ± 52.8	240.6 ± 53.4	146–391	0.002
Erythrocytes	10^6^/μl	4.66 ± 0.32	4.72 ± 0.31	4.0–5.8	0.056
Haemoglobin	g/dl	13.9 ± 1.0	14.1 ± 0.9	11.6–17.6	0.068
Haematocrit	%	40.3 ± 2.7	40.7 ± 2.6	34.6–50.6	0.163
MCH	pg	29.8 ± 1.1	29.8 ± 1.1	27–34	0.415
MCHC	g/dl	34.4 (32.5–37.1)	34.4 (32.7–36.0)	31.5–36.3	0.333
MCV	10^−9^μl	86.7 ± 3.4	86.3 ± 3.4	81–100	0.061

*Abbreviations: CRP* C-reactive protein, *C* cholesterol, *GOT* glutamic-oxaloacetic transaminase, *GPT* glutamic-pyruvic transaminase, *HDL* high-density lipoprotein, *IGF-1* insulin-like growth factor 1, *LDL* low-density lipoprotein, *MCH* mean corpuscular haemoglobin, *MCHC* mean corpuscular haemoglobin concentration, *MCV* mean corpuscular volume, *fT3* free triiodothyronine, *fT4* free thyroxine, *TC* total cholesterol, *TG* triglycerides, *TSH* thyroid stimulating hormone

### Complaints and adverse events via daily records

Additional file 1: Figure S1 reveals that a noticeably higher number of complaints were reported in the first week, peaking on day 4, when the subjects reported a total of 28 complaints. After day 7, the complaints levelled off and no serious adverse events were reported. The most predominant complaints over the study period were fatigue, headache, and diarrhoea (Additional file 1: Figure S2).

### Subjective physical sensations via questionnaire at POST

Additional file 1: Figure S3 shows the subjectively-rated physical sensations over the KD period assessed at POST. The most predominant physical sensation was a reduced feeling of hunger in 71.8% of the subjects. Almost half of the subjects reported decreased physical fitness. A third complained about the perception of less strength and peak power, respectively, whereas the majority (71.8%) reported having an unchanged endurance capacity. A total of 94.9% rated the KD’s implementation in daily life as easy or rather easy, and 87.2% claimed to be open-minded about repeating a KD.

## Discussion

The primary purpose of this controlled before-and-after comparison study was to evaluate the effects of a proven non-energy-restricted KD on physical performance, body composition, and blood parameters. Our diet intervention enjoyed very good compliance and was fully ketogenic, as verified by positive testing of urinary ketosis, dietary food records, and reduced RER at resting indicating increased lipid metabolism, respectively. Furthermore, our cohort was heterogeneous regarding age, gender, BMI, physical activity; moreover, their physical activity levels remained unchanged during the study period.

### Weight and body composition

Consistent with non-energy-restricted KD studies [12–14, 26], we observed mild weight loss over the entire 6-week KD period although mean energy intake did not change. Nevertheless, we cannot rule out the possibility that the 7-day food records of the last KD week were not representative for the whole KD period, and as most of the subjects reported feeling less hungry, a negative energy balance could have predominated during the KD’s first weeks. A negative energy balance could also be associated with the elevated excretion of energy-rich ketones via urine and breath [27]. Nordmann et al. showed that low-carbohydrate, non-energy-restricted diets are at least as effective as low-fat, energy-restricted diets in inducing weight loss for up to 1 year [28].

The results of both body composition assessments (ADP and BIA) were inconsistent regarding changes in FFM. However ADP, which is based on the same principles as the gold standard method of hydrostatic weighing [29], revealed that weight loss consisted in equal parts of reductions in FM and FFM. The unexpected decrease in REE, which contradicts the results of Alessandro et al. [30], could be partly explained by the slight decrease in FFM, the main determinant of REE [31]. We noted a significant positive correlation between FFM and REE (*r* = 0.749; *P* <0.001), but no relation between changes from PRE to POST in both parameters. This could indicate that the FFM loss did not comprise the metabolically active tissue compartment/muscles. This assumption is strengthened by the unaffected body cell mass, which represents the protein-rich and metabolically-active compartment [32], combined with an increased phase angle. The phase angle increase was due to a decrease in resistance R (reflecting fluid losses) and increase in reactance Xc (reflecting better cell membrane function). There is evidence that larger values are related to better outcomes in various diseases [33, 34]. Fluid loss could be related to the increased excretion of ketones and water in the urine during the state of ketosis [27]. The muscle-sparing effect during a metabolic state in which fatty acids are predominantly used as the energy source and the importance of sufficient protein intake during a KD are discussed in greater detail in a recent review by Paoli et al. [35].

Together with our result having documented a rise in hand grip strength as a surrogate marker of total muscle mass and function, we conclude that our intervention affected neither muscle mass nor muscle function negatively. The body composition changes may be regarded as positive.

### Physical performance

A study in 9 elite artistic gymnasts found no influence of a 4-week KD on explosive strength performance [12]. However, their study’s main limitation is that the authors defined their diet as being ketogenic despite a mean protein energy content of 41% and without having measured ketone bodies in blood or urine. As a high protein intake diminishes ketone production by favouring gluconeogenesis from abundant amino acids [36], it appears highly questionable that their diet was ketogenic. The few remaining studies that have investigated the impact of a non-energy-restricted KD on physical performance included a total of just 25 subjects with KD periods lasting 28 to 38 days [11, 13, 14].

Our primary outcome measure was VO_2_peak as measured during graded exercise to exhaustion, which reflects an individual’s aerobic physical fitness and is a key determinant of endurance capacity [37]. We found a mild but significant decrease in absolute VO_2_peak by 2.4% but the relative VO_2_peak (normalized to body weight) remained unchanged as the KD caused a decrease in body weight mainly based on FM and fluid loss. Phinney et al. [11] and Klement et al. [14] found that aerobic capacity was not compromised by a KD, while Zajac et al. [13] found a significant relative VO_2_peak improvement. This relative improvement should be interpreted with caution since the subjects’ body weight dropped significantly. Our subjects’ maximum work load (peak power) was comprised and decreased significantly by 4.1%, consistent with results others have reported [13, 14]. Zajac et al. [13] noted a significant (−3.3%; *P* = 0.037) and Klement et al. [14] a trend (−1.5%; *P* = 0.08) toward decreased peak power, respectively. This issue is further supported by the increased ratings of perceived exertion of our subjects after cycling test at POST, as such ratings correlate well with endurance performance [38]. In addition, our cohort’s subjectively-rated physical sensations that physical activities were more exhausting during the KD what was confirmed by the loss in peak power at POST. A major explanation therefore could be lower muscle glycogen stores combined with lower glycolytic-enzyme activity, which is compensated by enhanced capacity for fat oxidation and muscle glycogen sparing [39–41]. Investigations were therefore carried out to implement the KD’s beneficial metabolic adaptations to enhance fat oxidation in endurance sports by solving the muscle glycogen issue via short periods of carbohydrate intake, described in detail in a review [42].

In summary, our results reveal a slightly negative impact of a 6-week KD on physical performance. In the light of the importance of regular physical exercise during and after anti-cancer therapy [7], any diet that may compromise an individual’s capacity to adhere to an exercise regime would be of great concern, and raise the question of our findings’ clinical relevance. The KD impaired predominantly the endurance capacity and but not the performance in the submaximum area, as VT1 remained unchanged. In addition, activities of daily living and training in the aerobic zone would not be impaired. Further study is warranted to demonstrate the impact on endurance capacity of a longer KD period and after transition to normal diet after a KD.

### Blood parameters

All measured overnight fasting blood parameters at PRE and POST were within the reference ranges, but unexpectedly, approximately 40% of all parameters changed significantly. Our data and similar studies involving a non-energy-restricted KD in normal-weight, normolipidemic healthy adults confirm consistently significant increases in both total and LDL-C levels [13, 14, 43, 44] except for one study in 12 men reported decreased TG levels combined with unchanged TC, LDL-C and HDL-C levels after a 6-week KD [45]: they detected unchanged levels of HDL-C and TG and were in excellent agreement with an earlier study by Phinney et al. [44], but contrary to two other comparable studies reporting increased HDL-C and higher or lower TG levels [13, 14]. However, it is worth noting that all available comparable studies recruited small samples (8 to 12). A higher TG/HDL-C ratio has been identified as an index of the incidence and extent of cardiovascular disease and the incidence of type 2 diabetes mellitus [46] and this index decreased significantly during our study. A meta-analysis comparing the effects of non-energy-restricted low-carbohydrate (<60 g carbohydrates) vs. low-fat diets included five randomized controlled trials with a total of 447 obese subjects [28]. The authors concluded that long-term low-carbohydrate diets are associated with unfavourable changes in total and LDL-C levels, but favourable changes in TG and probably HDL-C levels. However, a 6-month non-energy-restricted KD intervention in 141 children with intractable seizures significantly increased TC, LDL-C, TG and atherogenic apoB-containing lipoproteins combined with a decrease in HDL-C, which corresponds to a potentially atherogenic blood lipid pattern [47].

There is evidence of strong associations between carbohydrate-restricted and fat-enriched diets and a significant increase in large LDL particles combined with a decrease in the number of small LDL particles [48, 49] and it has been hypothesised that large LDL particles have lower atherogenic potential [50]. Our subjects’ KD was rich in saturated fat (28% of energy) and the changes from PRE to POST of saturated fat intake and LDL-C correlated weakly (r = 0.294; *P* = 0.059) (data not shown). There is also evidence that a rise in saturated fat intake can elevate LDL-C levels [49]. Furthermore, Volek et al. showed that an energy-restricted KD rich in monounsaturated fat and supplemented with (n-3) fatty acids increased HDL-C and lowered TG levels [51]. As we did not assess lipoprotein subclasses, the atherogenic risk of our KD remains unclear and requires further investigation combined with the effect of a KD low in saturated but rich in monounsaturated fats.

Our subjects’ thyroid hormones changed significantly, but remained within the reference ranges. The impact of a KD on these hormones has only been marginally investigated. The sharp decline in the fT3 level and unchanged pituitary TSH in our subjects concur with those reports [14, 44, 52]. As Klement et al. [14] hypothesised, the KD-triggered increase in LDL-C is partly related to the significant drop in fT3 levels, as fT3 stimulates the expression of the LDL receptors on hepatic and peripheral cells, which are mandatory for LDL particle clearance [53]. The increase in fT4 combined with a decline in fT3 observed in our study is endorsed by a recent study in overweight men after a 4-week non-energy-restricted inpatient KD [54]. Probably due to the metabolic shift towards reliance on fatty acids for fuel at rest, and the less need for glucose uptake, we observed a significant decrease in fasting insulin, an observation consistently reported in the literature, namely large falls in the range of 34–48% during KDs [13, 45, 52, 55].

### Complaints and study limitation

Consistent with other studies [15, 56], our subjects complained about headache, gastrointestinal symptoms, and general weakness mainly during the 1-week metabolic adaptation phase to a KD. However, as the present study had no control group (a limitation), it remains unclear if the reported complaints were side effects directly related to the KD. Results from a non-controlled study should be interpreted with caution. Because of the high prevalence of thyroid dysfunction in our region 5 participants took thyroxin medication. As they had been taking this medication for over at least 6 months, we did not exclude these patients. Likewise, one participant taking methylphenidate and another inhaling daily glucocorticoids were not excluded due to stable medication for months. As we included healthy individuals, there was a broad range of leisure time activity. As we were not focusing on athletes, this diversity was intentional.

## Conclusions

In summary, our results reveal that a 6-week non-energy-restricted KD had a slightly negative impact on physical performance including endurance capacity, maximum work load, and faster exhaustion. Furthermore, we noted numerous metabolic adaptations, including alterations in many biochemical parameters. The significant weight loss, evenly distributed between FM and FFM, comprised neither muscle mass nor function. Our findings lead us to assume that a KD does not impact physical fitness in a clinically relevant manner that would impair activities of daily life and aerobic training. However, a KD may be a matter of concern in competitive athletes. Clearly, there is a need for longer-term trials focusing on lipoprotein subclass distributions and particle sizes. Studies are also required to evaluate the influence of the KD’s fatty acids composition on blood lipids.

## Additional file

Additional file 1: Table S1. Mean daily values from 7-days food records of diet compositions (energy, macronutrients, fatty acids, cholesterol, fibre and alcohol). **Table S2.** Mean daily values from 7-days food records of diet compositions (vitamins, minerals and trace elements). **Figure S1.** Sum of complaints/adverse events and number of subjects reporting these complaints via daily records during the study course. **Figure S2.** Itemised complaints/adverse events occurring during the complete study period. **Figure S3.** Subjectively-rated physical sensations over the KD period assessed at POST via a non-validated questionnaire. (PDF 638 kb)

## Abbreviations

ADP: Air displacement plethysmography
BIA: Bioelectrical impedance analysis
C: Cholesterol
FFM: Fat-free mass
FM: Fat mass
fT3: Free triiodothyronine
fT4: Free thyroxine
HDL: High-density lipoprotein
HDL-C: HDL cholesterol
HR: Heart rate
KD: Ketogenic diet
LDL: Low-density lipoprotein
LDL-C: LDL cholesterol
MET: Metabolic equivalent of task
POST: Last day of the study (day 42)
PRE: First day of the study
REE: Resting energy expenditure
RER: Respiratory exchange ratio
TC: Total cholesterol
TG: Triglycerides
TSH: Thyroid stimulating hormone
VO_2_peak: Peak oxygen uptake
VT1: Ventilatory threshold
W: Watt
