# Supplementary material for: Impact of a 6-week non-energy-restricted ketogenic diet on physical fitness, body composition and biochemical parameters in healthy adults
Source: Nutr Metab (Lond). 2017 Feb 20;14:17. doi: 10.1186/s12986-017-0175-5 (PMC5319032; doi:10.1186/s12986-017-0175-5)
Supplement: Additional file 1: Table S1. — Mean daily values from 7-days food records of diet compositions (energy, macronutrients, fatty acids, cholesterol, fibre and alcohol). Table S2. Mean daily values from 7-days food records of diet compositions (vitamins, minerals and trace elements). Figure S1. Sum of complaints/adverse events and number of subjects reporting these complaints via daily records during the study course. Figure S2. Itemised complaints/adverse events occurring during the complete study period. Figure S3. Subjectively-rated physical sensations over the KD period assessed at POST via a non-validated questionnaire. (PDF 638 kb) [file 12986_2017_175_MOESM1_ESM.pdf]

Additional File 1

Table S1 Diet compositions (energy, macronutrients, fatty acids, cholesterol, fibre and alcohol)

	Unit	Habitual diet	KD	*P*-value
**Energy, macronutrients, fibers and alcohol**				
Energy	kcal	2320.9 ± 551.3	2223.6 ± 583.7	0.186
Fat	g	99.4 ± 26.7	174.0 ± 52.6	<0.001
Fat	E %	41.0 ± 5.2	71.6 ± 5.0	<0.001
Carbohydrates	g	233.4 ± 69.9	40.4 ± 13.1	<0.001
Carbohydrates	E %	42.9 ± 6.5	7.7 ± 2.6	<0.001
Protein	g	75.0 (46.3 - 192.2)	105.7 (59.4 - 255.5)	<0.001
Protein	E %	15.0 (12.3 - 29.5)	20.9 (14.1 - 31.4)	<0.001
Fibre	g	22.3 (11.7 - 182.1)	21.2 (9.6 - 40.3)	0.053
Alcohol	g	5.7 (0 - 45.1)	0.5 (0 - 24.9)	<0.001
**Fatty acids and cholesterol**				
Saturated fats	g	41.0 ± 11.0	68.0 ± 22.0	<0.001
Saturated fats	E %	16.3 ± 3.0	27.9 ± 4.9	<0.001
Monounsaturated fats	g	32.7 ± 9.8	62.8 ± 20.0	<0.001
Monounsaturated fats	E %	13.0 ± 2.5	25.8 ± 4.3	<0.001
Polyunsaturated fats	g	12.39 (6.80 - 32.71)	22.25 (12.24 - 39.57)	<0.001
Polyunsaturated fats	E %	5.6 ± 1.8	9.8 ± 2.8	<0.001
Eicosapentaenoic acids	g	0.04 (0 - 0.37)	0.19 (0 - 0.57)	<0.001
Docosapentaenoic acids	g	0.12 (0 - 0.67)	0.32 (0.01 - 1.04)	0.001
Linoleic acid (n-6)	g	11.5 ± 4.5	17.6 ± 8.3	<0.001
Linolenic acid (n-3)	g	1.34 (0.56 - 13.24)	3.10 (1.03 - 11.80)	<0.001
Linoleic /linolenic acid ratio		8.1 ± 3.5	6.0 ± 3.0	0.003
Cholesterol	mg	321.2 ± 123.6	495.4 ± 167.0	<0.001

Abbreviations: n-3 = omega-3 fatty acid, n-6 = omega-6 fatty acid

Table S2 Diet compositions (vitamins, minerals and trace elements)

	Unit	Habitual diet	KD		*P*-value
**Fat-soluble vitamins**				**Reference range**	
Retinol equivalents	µg	1286.9 (500.1 - 7842.2)	1662.6 (764.4 - 4689.4)	800 - 1000	0.005
Vitamin D (calciferol)	µg	2.36 (1.09 - 7.56)	4.46 (1.80 - 13.50)	20	<0.001
Vitamin E (tocopherol)	mg	14.0 ± 5.4	18.5 ± 6.7	12	<0.001
Vitamin K	µg	129.0 (31.1 - 826.7)	147.4 (43.3 - 582.3)	60 - 70	0.639

**Water-soluble vitamins**					
Vitamin B1 (thiamin)	mg	1.14 (0.56 - 2.70)	1.17 (0.57 - 2.97)	1.0 - 1.2	0.247
Vitamin B2 (riboflavin)	mg	1.58 (0.86 - 5.23)	1.66 (1.02 - 3.43)	1.1 - 1.4	0.657
Vitamin B3 (niacin)	µg	28.7 (17.0 -66.6)	35.5 (17.1 -79.6)	12 - 15	0.035
Vitamin B5 (pantothenic acid)	mg	4.65 (2.41 - 13.50)	4.46 (2.43 - 9.94)	6	0.033
Vitamin B6 (pyridoxine)	mg	1.51 (0.70 - 3.60)	1.35 (0.68 - 3.34)	1.2 - 1.5	0.047
Vitamin B7 (biotin)	µg	50.8 (26.9 - 189.0)	45.5 (19.9 - 98.6)	30 - 60	0.003
Vitamin B9 (folic acid)	µg	261.6 (144.0 - 1146.7)	272.4 (159.4 - 575.6)	300	0.385
Vitamin B12 (cobalamin)	µg	4.47 (2.17 - 17.19)	6.81 (3.27 - 14.37)	3	<0.001
Vitamin C	mg	104.8 ± 45.4	97.9 ± 45.9	95 - 110	0.245
**Minerals and trace elements**					
Potassium	mg	3045.1 (1545.6 - 10131.6)	2456.2 (1429.4 - 4981.1)	2000	<0.001
Calcium	mg	1036.5 (402.0 - 2501.9)	1147.6 (682.4 - 2974.4)	1000	0.955
Magnesium	mg	398.7 (174.1 - 964.4)	316.7 (148.1 - 697.4)	300 - 350	<0.001
Phosphor	mg	1368.4 (698.6 - 2444.3)	1408.4 (782.6 - 2959.7)	700	0.520
Iodine	µg	101.2 ± 30.8	121.4 ± 60.3	200	0.060
Iron	mg	12.7 (5.1 - 61.2)	9.0 (4.8 - 19.5)	10 - 15	<0.001
Zinc	mg	11.7 ± 3.4	12.0 ± 3.5	7 - 10	0.597
Selenium	µg	0.14 (0 - 18.3)	0 (0 - 33.3)	60 - 70	0.016

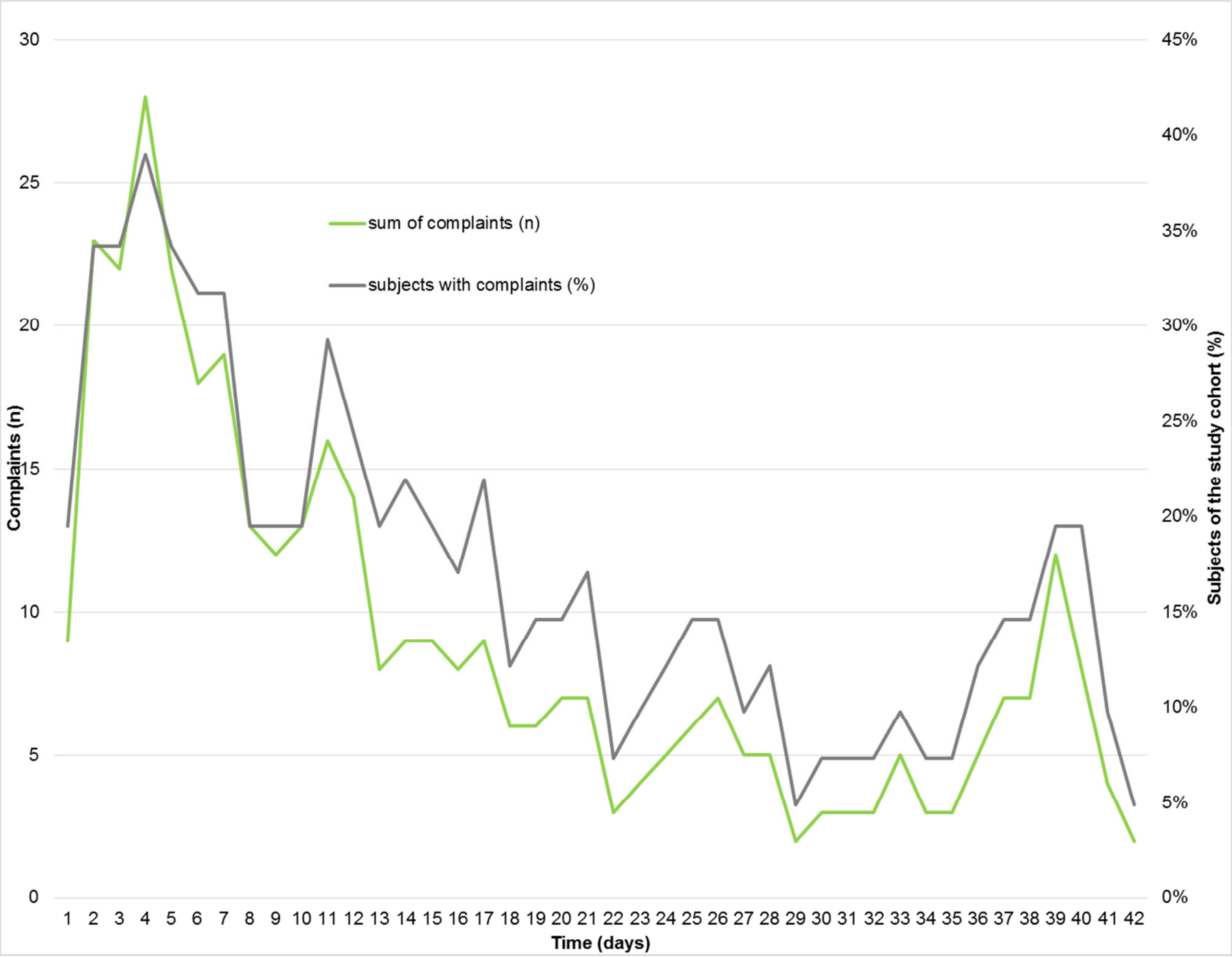

Figure S1 Sum of complaints/adverse events and number of subjects reporting these complaints via daily records during the study course

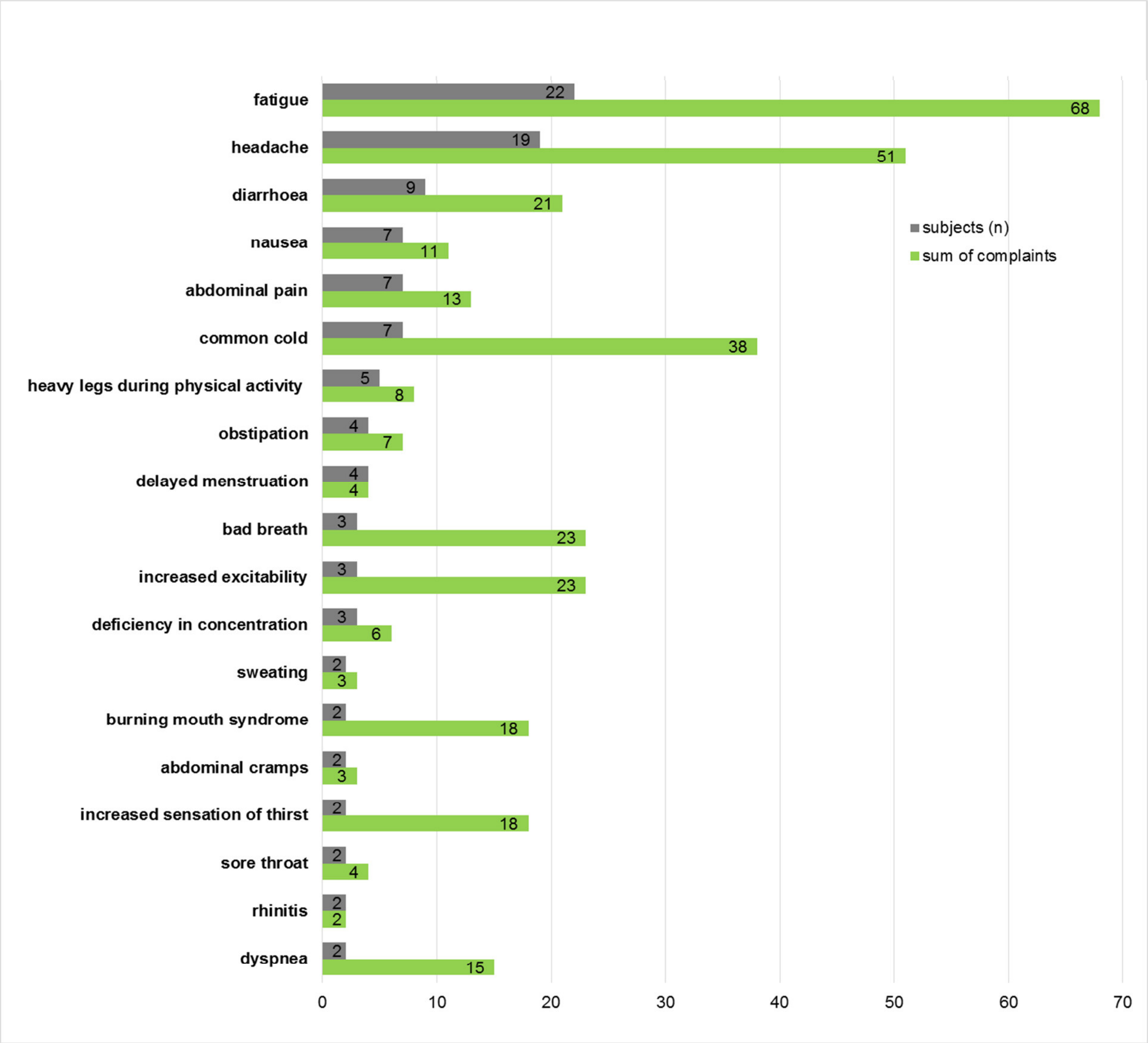

Figure S2 Itemised complaints/adverse events occurring during the complete study period.

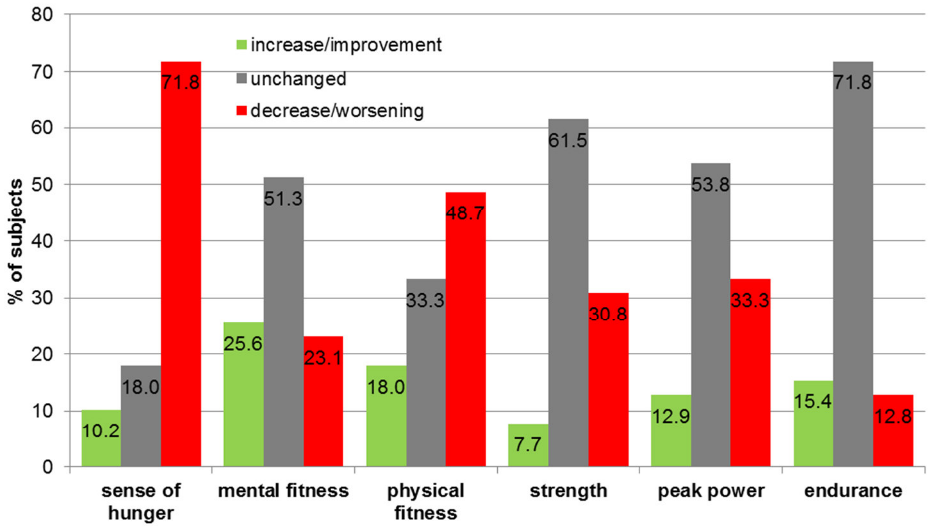

Figure S3 Subjectively-rated physical sensations over the KD period assessed at POST via a non-validated questionnaire.
